# Significance of *LHCGR* polymorphisms in polycystic ovary syndrome: an association study

**DOI:** 10.1038/s41598-023-48881-0

**Published:** 2023-12-21

**Authors:** Sukhjashanpreet Singh, Mandeep Kaur, Archana Beri, Anupam Kaur

**Affiliations:** 1 Department of Human Genetics, Guru Nanak Dev University, Amritsar, Punjab 143005 India; 2 Beri Maternity Hospital, Southend Beri Fertility and IVF, Amritsar, Punjab 143001 India

**Keywords:** Genetics, Endocrinology

## Abstract

This study was conducted to analyze the association of Luteinizing Hormone/Choriogonadotropin Receptor (*LHCGR*) gene rs4953616 and rs7371084 polymorphisms with the risk of polycystic ovary syndrome (PCOS) in Punjab, India. A total of 823 women (443 PCOS cases and 380 healthy controls) were enrolled in the present study. The polymerase chain reaction-restriction fragment length polymorphism technique (PCR–RFLP) was used for genotyping. Anthropometric parameters, lipid and hormonal profiles, were compared between the two groups. Demographic features were compared using Mann Whitney U test while the Chi-square test and odds ratios (ORs) were used to assess the genetic association and risk towards PCOS, respectively. A one-way analysis of variance (ANOVA) test was employed to analyze the correlation of genotypes with baseline parameters in PCOS cases. A statistically significant difference was revealed in the genotypic and allelic frequencies of rs4953616 polymorphism between PCOS cases and controls (p = 0.01 and p = 0.004, respectively). The mutant genotype (TT), mutant allele (T), and recessive model of rs4953616 polymorphism conferred 1.77, 1.3, and 1.5 times risk towards PCOS, respectively. No significant distribution for genotypes and alleles was found for rs7371084 in both groups (p = 0.25 and p = 0.26, respectively). In addition to dyslipidemia, PCOS women also had significantly higher body mass index (BMI) and waist-to-hip ratio (WHR), testosterone (T), and luteinizing hormone (LH). Upon haplotype analysis, the TT haplotype was found to be significantly associated with the increased risk of PCOS. Our results demonstrated a significant role of *LHCGR* rs4953616 polymorphism in the development of PCOS.

## Introduction

PCOS is the most prevalent endocrine-metabolic condition in women of fertile age with a global prevalence of 5% to 20%^1,2^. The variation in the prevalence of PCOS could be due to the heterogeneity in ethnicity and age distribution in the population from which data has been collected^3^. During the 2003 Rotterdam Consensus Workshop, PCOS was defined as a multi-system matrix of anomalies including obesity, hyperinsulinemia, menstrual irregularities, hyperandrogenism, small cysts in one or both ovaries, and elevated concentrations of LH^4^. In addition, PCOS has also been linked to impaired glucose tolerance, increased risk of type 2 diabetes, endometrial cancer, cardiovascular diseases, dyslipidemia, and depression which further worsen the quality of life^5,6^. Several hypotheses have been proposed over time to pinpoint the precise underlying processes that lead to the development of PCOS. However, due to its heterogeneous nature and uncertain etiology, by the time a diagnosis is made, PCOS manifests as a self-perpetuating cycle of multiple system dysfunction^7^. Family studies and genome-wide association studies (GWAS) have supported the contribution of genetic factors to the pathophysiology of PCOS^8,9^. The phenotype of PCOS, diagnostic criteria employed, and racial background, all influence the narrowing down of the PCOS susceptibility loci^10^. However, a few genetic loci that were found to be associated with PCOS development include *LHCGR*, *FSHR*, *THADA,* and *DENND1A* and the interaction among these loci and multiple proteins under the influence of environmental factors affects PCOS progression^11^.

The LHCGR is directly involved in the orchestrated series of events that regulate normal sexual maturation and fertility in males and females^12^. LHCGR acts as a high-affinity receptor for LH and upon binding leads to steroidogenesis, follicular development, and formation of corpus luteum^13^. The LHCGR belongs to the G-protein coupled receptor family, and in women, is primarily expressed in ovarian theca cells and differentiated granulosa cells^14,15^. In contrast to theca cells which constitutively express LHCGR, granulosa cells express LHCGR only at the later stages of follicular development after they become fully differentiated. Induction of LHCGR allows the preovulatory follicle to respond to the mid-cycle surge of LH with ovulation^13^.

The *LHCGR* gene is mapped on chromosome 2 (2p16.3) comprising 11 exons that code for 675 amino acid trans-membrane glycoprotein^16^. In GWAS, several polymorphisms in the *LHCGR* have been reported and these SNPs may alter the gene expression or protein function influencing its biological activity which causes typical features of PCOS in women of reproductive age^17,18^. The studies that investigated the association of susceptible SNPs across the globe have come up with strong evidence for the association of *LHCGR* polymorphisms with PCOS despite conflicting results^19–22^.

The rs13405728 intronic polymorphism was reported as the most significant and found to be associated with PCOS in Chinese^23^. However, in European ancestry, rs13405728 was not associated with the risk of PCOS^17^. Our previous study demonstrated the crucial role of rs2293275 and rs12470652 polymorphisms in the development of PCOS^22^. A study on Bahraini Arab women was the first to report the association of novel rs4953616 and rs7371084 SNPs with PCOS^24^. All these inconsistencies in the results of different association studies could be attributed to ethnic disparities and the availability of different diagnostic criteria.

The present study aimed to evaluate the association of *LHCGR* rs4953616 and rs7371084 polymorphisms with a risk of PCOS in the Punjabi population.

## Material and methods

### Sample collection and biochemical evaluation

The study was approved by the Ethics Review Board of Guru Nanak Dev University, Amritsar (297/HG) which is in accordance with the Declaration of Helsinki. A total of 823 women were enrolled in the present study, 443 of whom were diagnosed with PCOS and 380 age-matched healthy controls with no signs of PCOS. Women with PCOS were recruited from Beri Maternity Hospital, Amritsar, Punjab, India after the confirmed diagnosis by a gynecologist. Informed consent was obtained from all the participants followed by a withdrawal of 5 mL peripheral blood from each case and control, 3 mL was transferred to a vacutainer containing 0.5 M EDTA for molecular assays and 2 mL was poured into the vacutainer containing clot activator for biochemical analysis. The predesigned proforma was used to collect the relevant information like demographics, reproductive history, family history, and pedigree. The included subjects' anthropometric data including height, weight, WHR, and BMI was also obtained. The inclusion and exclusion criteria for the selection of both groups have been described in our previous study^25^. The 2 mL blood was used for serum extraction which was then subjected to biochemical analysis including measurements of total cholesterol, high-density lipoprotein (HDL), and triglycerides levels, and specific kits provided by Erba Mannheim were used for the analysis. Friedewald’s formula was used to estimate the proportion of low-density lipoprotein (LDL) and very low-density lipoprotein (VLDL)^26^. The levels of T, LH, and follicle-stimulating hormone (FSH) were also measured using Calbiotech’s ELISA kits.

### DNA extraction and genotyping

Genomic DNA was isolated from the 3 ml of blood using the phenol–chloroform method^27^. Nanodrop and agarose gel electrophoresis were used to assess the quality and concentration of isolated DNA. For genotype rs4953616, an amplification refractory mutation system (ARMS) PCR technique was used. Tetra primers (2 outer primers and 2 inner primers) were designed using the Primer 1 software to amplify the target region (http://primer1.soton.ac.uk/primer1.html) and then 1.8% ethidium bromide-stained gel was used to visualize the amplified products. The bands of 406 bp and 185 bp represented the CC homozygous genotype, bands of 406 bp and 268 bp indicated the TT homozygous genotype whereas the bands of 406 bp, 185 bp, and 268 bp signified CT heterozygous genotype. The PCR–RFLP technique was performed to genotype the rs7371084 polymorphism. The amplification was carried out using a specific set of primers which were designed using the Primer 3 tool (https://bioinfo.ut.ee/primer3-0.4.0/) to obtain a 315 bp product followed by restriction digestion using the HaeIII enzyme (New England Biolabs) at 37 °C for 2 h. After that, RFLP products were electrophoresed on 2.2% agarose gel and the band of 315 bp indicated wild-type genotype (TT), bands of 315 bp, 229 bp and 86 bp signified heterozygous genotype (TC) and bands of 229 bp and 86 bp represented homozygous mutant genotype (CC). Details of the primers used for amplification are given in Table 1.

**Table 1:** Primer sequence and annealing temperature.

Polymorphism	Primer set	5′–3′ sequence	Annealing temp
rs4953616	Forward inner	ATCCTCATCATCATTTCCATTATCCC	65.5 °C
	Reverse inner	TGAGGGTTAGGCTTCTTGGCA	
	Forward outer	TCCATATAGTTTGCTGAAGGAGGGA	
	Reverse outer	TTTCAACACCCAGAAAACCCATATG	
rs7371084	Forward	GCCAGGCACAGCGTTAAG	63 °C
	Reverse	GCTGTCAGTCTGAAGTTGGG	

### Statistics

The continuous variables were checked for normal distribution using the Kolmogorov test and as variables did not follow normal distribution Mann Whitney test was applied and results were given as median and interquartile range and a p-value less than 0.05 was considered significant. CaTS–Power Calculator was used to evaluate the power of the study and sample size indicating power to be 82.5% with a confidence interval (CI) of 95%. The Hardy–Weinberg equilibrium (HWE) was applied to all the controls for rs4953616 and rs7371084 polymorphisms and it was found that both of the SNPs were significantly following the HWE. The Statistical Package for Social Sciences (version 21, IBM SPSS, NY, USA) was used to analyze the data. The chi-square test was performed to evaluate the significant distributions of genotypic and allelic frequencies of both the SNPs among PCOS cases and controls. To quantify the risk of each polymorphism for the development of PCOS, ORs were calculated using MedCalc software. The biochemical variables, anthropometric features, and hormonal characteristics were compared concerning genotypes by performing an ANOVA test. Haplotype analysis was carried out using Haploview version 4.2 software. Bonferroni correction was adjusted for genetic analysis and ANOVA tests.

## Results

### Baseline characteristics of the study participants

The median age for PCOS women in this case–control study was 24, whereas 23 for healthy controls as both groups were age-matched, no significant difference was detected. However, in the studied population, women with PCOS appeared to be experiencing early-age menarche as compared to control women (p = 0.03). Dyslipidaemia was associated with PCOS women with significantly elevated cholesterol (p < 0.0001), triglycerides (p < 0.0001), LDL (p = 0.04), and VLDL (p < 0.0001) levels, and lower HDL levels compared to control women (p < 0.0001). The levels of T (p < 0.0001) and LH (p = 0.01) were raised whereas FSH levels were consistent between both cohorts following the hormonal evaluation. BMI and WHR showed significantly higher values in PCOS females (p < 0.0001) (Table 2).

**Table 2:** Comparison of demographic and biochemical characteristics between both groups.

Variables	Cases (N = 443)	Controls (N = 380)	p-value
Age	24 (21–27)	23 (21–26)	0.31
Age at Menarche^a^	13 (9–16)	13 (10–16)	0.03*
Obesity-related parameters			
BMI	23.3 (20.8–26.6)	21.6 (19.6–24.3)	< 0.0001*
WHR	0.89 (0.85–0.93)	0.82 (0.78–0.88)	< 0.0001*
Lipid profile			
Cholesterol	168.9 (145–200)	154.2 (136–179.4)	< 0.0001*
Triglycerides	140 (93.5–193.8)	96.7 (55.5–134.7)	< 0.0001*
HDL	43.1 (36.8–50.2)	46 (39.4–56.3)	< 0.0001*
LDL-C	93.3 (67.2–126.5)	86.1 (67.7–111.6)	0.04*
VLDL-C	27.9 (18.4–38.4)	19.3 (11.1–26.9)	< 0.0001*
Hormonal profile			
LH	7.2 (4.5–10.2)	5.1 (2.9–6.5)	0.01*
FSH	5.6(4.1–7.6)	5.8 (4.7–9)	0.69
LH/FSH	1.06 (0.69–1.9)	0.83 (0.54–1.6)	0.06
Testosterone levels	0.84 (0.67–1.11)	0.44 (0.36–0.72)	< 0.0001*

N: no of individuals; BMI: Body Mass Index; WHR: waist-hip-ratio; HDL: high-density lipoprotein; LDL: low density lipoprotein; VLDL: very low-density lipoprotein; LH: luteinising hormone; FSH: follicle stimulating hormone, Mann Whitney U test (Interquartile range).

*p-value considered significant < 0.05.

^a^Maximum and minimum percentile were used instead of interquartile.

### Genotype analysis

The frequency distribution of genotypes and alleles for rs4953616 polymorphism was statistically significantly different among both of the groups (p = 0.01 and p = 0.004, respectively). The carriers of mutant genotype (TT) and mutant allele (T) were at 1.77 (OR: 1.77; CI 1.19 to 2.63; p = 0.004) and 1.3 (OR: 1.3; CI 1.09 to 1.61; p = 0.004) times more risk to develop PCOS, respectively. The recessive model for rs4953616 polymorphism was also revealed to confer a 1.5-fold risk for PCOS progression (p = 0.005). On the contrary, no significant genotypic and allelic frequency distribution was found for rs7371084 in cases and controls (p = 0.25 and p = 0.26, respectively) (Table 3).

**Table 3:** Distribution of genotypic, allelic frequencies, and genetic models of rs4953616 and rs7371084 polymorphisms between PCOS cases and controls.

SNP	Genotype/allele	Case (%)	Control (%)	χ^2^ p-value	OR (95% CI)	p-value
rs4953616	CC	92 (21)	100 (26.3)	**1.0E**^−2^*	Reference	–
	CT	217 (49)	198 (52.1)		1.19 (0.84 to 1.67)	0.3
	TT	134 (30)	82 (21.6)		1.77 (1.19 to 2.63)	**4.0E**^−3^*
	C	401 (45.3)	398 (52.4)	**4.0E**^−3^*	Reference	–
	T	485 (54.7)	362 (47.6)		1.3 (1.09 to 1.61)	**4.0E**^−3^*
Dominant model	TT + CT vs. CC	351/92	280/100	0.06	1.3 (0.98 to 1.88)	0.06
Recessive model	TT vs. CT + CC	134/309	82/298	**4.0E**^−3^*	1.5 (1.14 to 2.16)	**5.0E**^−3^*
Heterozygous model	CT vs. TT + CC	217/226	198/182	0.3	0.8 (0.67 to 1.16)	0.3
rs7371084	TT	397 (89.6)	329 (86.5)	0.25	Reference	–
	TC	42 (9.4)	49 (13)		0.7 (0.45 to 1.09)	0.1
	CC	4 (1)	2 (0.5)		1.6 (0.30 to 9.10)	0.5
	T	836 (94.4)	707 (93)	0.26	Reference	–
	C	50 (5.6)	53 (7)		0.7 (0.53 to 1.18)	0.2
Dominant model	CC + TC vs. TT	46/397	51/329	0.1	0.74 (0.48 to 1.14)	0.17
Recessive model	CC vs. TC + TT	4/439	2/378	0.5	1.7 (0.31 to 9.45)	0.5
Heterozygous model	TC vs. CC + TT	42/401	49/331	0.1	0.7 (0.45 to 1.09)	0.12

χ^2^: chi-square; OR: odds ratio.

*p-value considered significant after Bonferroni correction.

### Multiple comparisons analysis

The ANOVA test was conducted to evaluate the possible impact of individual genotypes on BMI, WHR, lipid profile, and hormonal parameters of PCOS for both polymorphisms in cases. The study findings indicated a marginal but not significant increase in the levels of triglyceride (p = 0.08), VLDL (p = 0.16) (Table 4), and T (p = 0.16) (Table 5) among women with PCOS who carried the CC genotype of the rs7371084 polymorphism.

**Table 4:** Comparison of means of biochemical and anthropometric characteristics concerning genotypes in PCOS cases (mean ± SD).

	rs4953616				rs7371084			
	CC	CT	TT	p-value	TT	TC	CC	p-value
BMI	24.56 ± 4.63	23.53 ± 4.70	24.14 ± 4.05	0.14	23.86 ± 4.46	24.52 ± 4.86	24.0 ± 5.96	0.67
WHR	0.89 ± 0.04	0.87 ± 0.06	0.88 ± 0.06	0.06	0.883 ± 0.06	0.885 ± 0.07	0.885 ± 0.06	0.97
Cholesterol	175.75 ± 46.11	177.58 ± 50.95	172.68 ± 52.11	0.67	175.75 ± 50.74	177.04 ± 45.09	158.75 ± 66.84	0.78
Triglyceride	164.24 ± 93.97	150.82 ± 79.27	166.21 ± 94.50	0.21	155.51 ± 85.45	177.96 ± 101.9	224.82 ± 76.42	0.08
LDL	97.70 ± 46.52	104.17 ± 52.62	93.89 ± 57.24	0.19	99.94 ± 53.96	100.15 ± 47.19	73.16 ± 73.0	0.60
VLDL	32.02 ± 18.09	29.78 ± 15.44	33.22 ± 18.89	0.17	30.89 ± 16.89	33.79 ± 19.14	44.95 ± 15.25	0.16
HDL	46.02 ± 13.42	43.62 ± 13.39	45.56 ± 12.29	0.22	44.91 ± 13.25	43.10 ± 11.91	40.63 ± 7.75	0.57

One-way ANOVA.

**Table 5:** Comparison of means of hormonal characteristics concerning genotypes in PCOS cases (mean ± SD).

	rs4953616				rs7371084			
	CC	CT	TT	p-value	TT	TC	CC	p-value
LH	8.66 ± 4.15	8.32 ± 4.47	6.83 ± 4.51	0.42	8.07 ± 4.30	9.36 ± 6.68	3.73 ± 0.68	0.33
FSH	7.16 ± 2.64	5.95 ± 2.58	5.52 ± 3.31	0.21	6.12 ± 2.94	7.17 ± 1.91	6.22 ± 2.10	0.83
LH/FSH	1.29 ± 0.74	1.68 ± 1.40	1.91 ± 2.14	0.48	1.68 ± 1.54	1.54 ± 1.37	0.61 ± 0.09	0.62
Total testosterone	0.74 ± 0.39	0.59 ± 0.26	0.75 ± 0.28	0.16	0.68 ± 0.31	0.49 ± 0.14	1.04 ± 0.008	0.16

One-way ANOVA.

### Haplotype analysis

**Figure 1:**
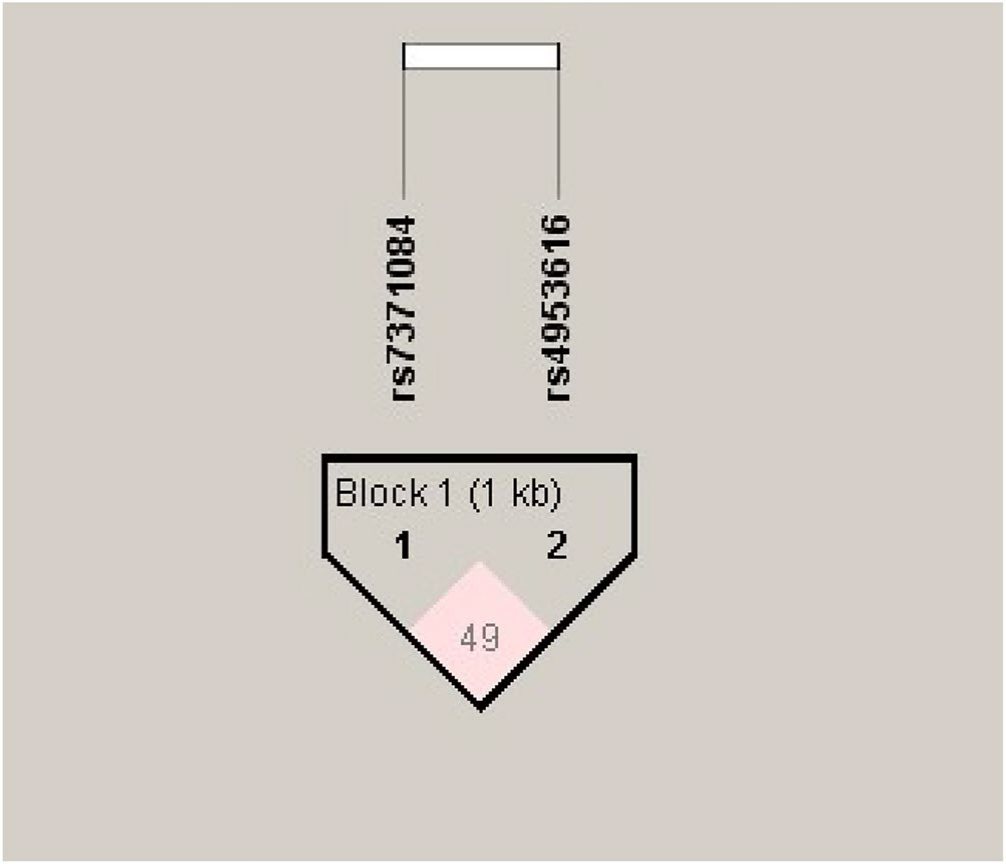
Combined LD plot (PCOS cases and controls) for *LHCGR* polymorphisms.

The Haploview version 4.2 software was used to analyze the haplotypes of *LHCGR* rs4953616 and rs7371084 polymorphisms in cases and controls. Both the SNPs appeared to be moderately in linkage disequilibrium (LD) (D′ = 0.498, LOD = 2.86, r^2^ = 0.016) (Fig. 1). We observed the TT haplotype to be significantly associated with an increased risk of PCOS (OR: 1.40; CI 1.04 to 1.85; p = 0.02) (Table 6).

**Table 6:** Haplotype analysis of rs4953616 and rs7371084 polymorphisms.

Haplotypes	Case frequency (N)	Control frequency (N)	OR (95% CI)	p-value
CT	0.439 (194)	0.509 (193)	–	–
TT	0.505 (224)	0.421 (160)	1.40 (1.04 to 1.85)	**2.0E**^−2^*
TC	0.043 (19)	0.054 (21)	0.90 (0.46 to 1.72)	0.75
CC	0.014 (6)	0.016 (6)	0.99 (0.31 to 3.13)	0.99

OR: odds ratio; CI: confidence interval.

*p-value considered significant after Bonferroni correction.

## Discussion

Association studies have always been valuable for determining the putative genetic loci that correlate to disease risk, and the significance of these studies dramatically increased in multifaceted disorders like PCOS where several loci from different pathways are involved in the pathogenesis of the syndrome. By conducting association analyses researchers may accurately narrow down the causal SNPs believed to be linked with the onset or progression of the disease. The present study comprised of 823 women was conducted to evaluate the association of *LHCGR* rs4953616 and rs7371084 polymorphisms with the risk of PCOS development. In the studied population, women with PCOS experienced early menarcheal age as compared to non-PCOS women (p = 0.015). However, our findings are in disagreement with the studies conducted on Colombian and Bahraini Arab women (p = 0.18 and p = 0.24, respectively)^24,28^. In this study, we found dyslipidemia to be significantly associated with PCOS cases (p < 0.015). Our findings were supported by study on Chinese women revealing 41.3% of women had dyslipidemia^29^. Another study from China demonstrated the high prevalence of dyslipidemia to be associated with PCOS women and the most prevailing factor was low HDL levels^30^. However, our findings are in disagreement with the study from Dubai^31^. A study from Mardan, Pakistan reported an association of abnormal lipid profiles with anxiety and depression in women with PCOS^32^. The present study demonstrated higher values of BMI and WHR in PCOS women (p < 0.0001). Our results are in agreement with the study from Madhya Pradesh^33^ and Maharashtra, India^34^. On the contrary, a study conducted by Kaluzna et al. on Polish females found no significant difference in WHR between both groups^35^. A significant association of high BMI in women with PCOS was also documented in the Pakistani population^36^. Another significant association between high BMI and WHR values and PCOS was reported by a Northeast Indian study^37^.

Among the hormonal parameters, LH (p = 0.01) and T (p < 0.0001) concentrations were significantly elevated in women with PCOS as compared to controls. We also evaluated the correlation between genotypes of both polymorphisms and hormonal profiles. No significant association was observed between any genotype of rs4953616 and rs7371084 polymorphisms and hormones. This is in contrast with the findings published on Arab women where researchers demonstrated rs4953616 to be negatively associated with T although no association of rs7371084 with hormones (LH, FSH, and T) was observed^24^. On the contrary, another study illustrated the increased LH levels (p = 0.02) in an overdominant model (TT + CC) of rs7371084 in individuals with PCOS, however, results for rs4953616 were in agreement with our findings^28^.

Our study found a statistically significant difference in the genotypic (p = 0.01) and allelic frequencies (p = 0.004) for rs4953616 polymorphism upon comparing cases and controls. It was revealed that the mutant genotype (TT) and mutant allele (T) conferred 1.77 (CI 1.19 to 2.63; p = 0.004) and 1.3 (CI 1.09 to 1.61; p = 0.004) folds risk to the PCOS development. We also observed that among the genetic models, the recessive model provided 1.5 (CI 1.14 to 2.16; p = 0.005) times more risk of PCOS (Table 3). Our findings are supported by a study on Bahraini Arab women demonstrating rs4953616 polymorphism to be associated positively with PCOS^24^. However, the present study is in disagreement with a pilot study conducted on Colombian women^28^. In contrast, no significant difference in frequency distribution is found for rs7371084 polymorphism. However, Almawi et al. (2015) found a negative association between rs7371084 and PCOS^24^. Similar findings were delivered by Alarcón-Granados et al. where they reported rs7371084 polymorphism to be linked negatively with PCOS in the dominant, overdominant, and codominant models^28^.

Both the SNPs were subjected to haplotype analysis which resulted in moderate LD between the two polymorphisms (D′ = 0.498 and r^2^ = 0.016). A similar trend of LD for rs4953616 and rs7371084 was reported by Almawi et al.^24^. However, complete LD (D′ = 1) and low recombination (r^2^ = 0.08) were illustrated in a pilot study on Colombian women^28^. Our study also revealed a significant difference in the distribution of haplotypes among both cohorts and the occurrence of haplotype TT was found to be associated with an increased risk of PCOS (OR: 1.40; CI 1.04 to 1.85; p = 0.02) (Table 6). These findings were found to be in disagreement with other studies on Bahraini Arab and Colombian women^24,28^.

## Conclusion

The present case–control study demonstrated the association of *LHCGR* rs4953616 polymorphism with PCOS in the Punjabi population. Genetic association studies have been proven to uncover the potential genes or genomic regions and elucidate novel pathways that make a contribution to certain disease or trait. Further research on larger scale including populations from related or distinct ethnic groups is required to validate the association of *LHCGR* polymorphisms with PCOS. The data can be utilized to facilitate the advancement of therapeutic interventions, and screening purposes to take preventive measures before onset of disease.

## Data Availability

The manuscript contains all the data which have been collected and analyzed during the course of this study.
